# Seroprevalence of SARS-CoV-2 antibodies in Republic of Congo, February 2022

**DOI:** 10.1017/S0950268823001425

**Published:** 2023-09-11

**Authors:** Gilbert Ndziessi, Roch Fabien Niama, Axel Gilius Aloumba, Jethro Massala Peya, Joseph Axel Ngatse, Ryschel Alist Ngoyomi, Ange Clauvel Niama, N’Kaya Tobi, Antoine Loussambou, Jean Medard Kankou, Benjamin Atipo, Jean Claude Emeka, Pascal Ibata, Donatien Moukassa, Alexis Elira Dokekias

**Affiliations:** 1 Department of Public Health, Faculty of Health Sciences, Marien Ngouabi University, Brazzaville, Republic of the Congo; 2 National Laboratory of Public Health, Brazzaville, Republic of the Congo; 3 Department of Infectious Diseases, University Hospital of Brazzaville, Brazzaville, Republic of the Congo; 4 Ministry of Public Health and Population, Brazzaville, Republic of the Congo; 5 Ministry of Agriculture, Fisheries and Livestock, Brazzaville, Republic of the Congo; 6 Army Hospital, Brazzaville, Republic of the Congo; 7 Clinical and Molecular Biochemistry Unit, Faculty of Health Sciences, Marien Ngouabi University, Brazzaville, Republic of the Congo; 8 Department of Medicine, Faculty of Health Sciences, Marien Ngouabi University, Brazzaville, Republic of the Congo

**Keywords:** antibodies, Congo, COVID-19, seroprevalence, SARS-CoV-2

## Abstract

In resource-limited countries, the lack of widespread screening masks the true situation of COVID-19. We conducted this study to assess SARS-CoV-2 spread by detection of specific antibodies and to determine associated factors. A population-based cross-sectional study was conducted. Subjects were tested for the presence of two antibodies (IgM and IgG) specific to SARS-CoV-2. Data collection was done using a smartphone with the KoboCollect application. Prevalence of antibodies was estimated with 95% confidence intervals. Logistic regression was used to determine factors associated with positive serological test. A total of 9,094 persons were tested in 4,340 households. The mean age was 30.18 ± 18.65 years, 46.5% male. The overall seroprevalence (prevalence, 95% CI) of SARS-CoV-2 antibodies was (48.2% [47.2%–49.2%]). Being vaccinated, having been in contact with a COVID-19 patient, being older than 50 years, living in a union, having secondary education and having tertiary education were factors independently associated with the likelihood of having anti-sars-CoV-2. We estimate in February 2022 that 48% persons had antibodies against the COVID-19 virus, more among those vaccinated. Vaccination intensification in low prevalence departments will reduce the risk of new outbreaks.

## Introduction

COVID-19 was first reported in China in the Hubei province of Wuhan in December 2019. It has rapidly evolved into a pandemic [1]. According to the global epidemiological situation on 10 July 2022, there are 552,504,624 cases and 6,347,816 deaths, representing a case fatality rate of 1.1%. The hecatomb predicted in Africa due to health system failure never occurred, as the Africa Centres for Disease Control and Prevention (Africa CDC) reported 11,994,754 cases of COVID-19 in the continent, including 255,127 deaths for a case-fatality rate of 2.1% until 1 August 2021 [2]. Polymerase chain reaction (PCR) for detection of viral nucleic acid in upper and lower respiratory tract specimens is the gold standard for clinical diagnosis of COVID-19, but its supply is limited in African countries [3, 4]. In this context, the number of positive cases based on PCR diagnosis does not predict the level of SARS-CoV-2 circulation in the population.

In the case of a new coronavirus, the initial seroprevalence in the population is assumed to be negligible, due to the new origin of the virus. Therefore, monitoring of antibody seropositivity in a population may allow conclusions to be drawn about the extent of infection and the cumulative incidence of infection in the population. Studies conducted regarding the circulation of SARS-CoV-2 in some African countries revealed prevalence ranging from 19.1% to 65.0% in the general population [5–16] and 4.0% to 63.0% in specific populations [17–24]. Results of a recent study using a meta-analysis of proportions show that the overall seroprevalence of anti-SARS-CoV-2 antibodies in Africa was 16.0% (95% confidence interval [CI]: 13.1–18.9%) in the period 2020–2021 [25]. Based on these statistics, it is safe to say that the virus has spread widely in African countries, in contrast to the estimations published by governments [2].

Congo reported its first case of COVID-19 on 14 March 2020 and started vaccination on 24 March 2021. As of 31 January 2022, 350,775 people were tested, 23,730 were positive for SARS-CoV-2, and the positivity rate was 6.7%. A total of 695,665 people received at least one dose of vaccine, which represents vaccination coverage of 12% in February 2022 [26]. The target of the COVID-19 vaccination in Congo, which includes persons over 15 years of age, represents 60% of the population, that is, 3,468,907 persons corresponding to the target level for achieving herd immunity. So, 2,773,242 people have not received any doses of vaccine. We note that most of the population were not vaccinated against COVID-19 and few people were screened. In this context, the following questions can be asked: what is the proportion of people who have antibodies against SARS-CoV-2? What is this proportion among vaccinated people and among unvaccinated people? Answering these questions allows us to assess the level of SARS-CoV-2 circulation in this country. To our knowledge, no study has been conducted to assess the national seroprevalence of SARS-CoV-2 in Congo. The objective of this study was to determine, two years after the first case of COVID-19 was reported in Congo, the proportion of people with antibodies against SARS-CoV-2 distributed by age and department and to identify the factors associated with positive serology. These results will contribute to reorienting the response strategy against COVID-19 at the country level, particularly in terms of vaccination.

## Methods

### Design and period of study

A cross-sectional study was conducted in the general population. Data collection took place from 8 to 28 February 2022.

### Databases

The database of the last general population and housing census of 2007 (GPHC 2007) available at the National Institute of Statistics was used. This database contains information on 4,143 counting zones (CZ), including their geographical location, number of households, and population. The CZ is the smallest geographical statistical unit created during the census of population and housing. Table 1 shows the tree structure of the database: a first structure based on the distribution of CZ, a second structure defined by the distribution of households, and a third structure based on the distribution of the population.

**Table 1.** Distribution of counting zones, households and population by department and area of residence

Area of residence	Department	Number of counting zones (CZ)	%	Number of households	%	Population	%
Urban	Niari	130	0.031	23,049	0.026	97,035	0.026
	Bouenza	67	0.016	16,938	0.019	71,620	0.019
	Sangha	30	0.007	6,423	0.007	28,178	0.008
	Brazzaville	1,335	0.322	328,598	0.371	1,373,394	0.371
	Pointe-Noire	696	0.168	172,657	0.195	715,327	0.193
	Subtotal	2,258	0.545	547,665	0.619	2,285,554	0.618
Rural	Kouilou	138	0.033	25,626	0.029	91,958	0.025
	Niari	179	0.043	30,726	0.035	134,240	0.036
	Lékoumou	129	0.031	21,366	0.024	96,400	0.026
	Bouenza	299	0.072	58,427	0.066	237,453	0.064
	Pool	312	0.075	66,846	0.076	236,592	0.064
	Plateaux	241	0.058	39,203	0.044	174,591	0.047
	Cuvette	226	0.055	35,619	0.040	156,042	0.042
	Cuvette-Ouest	122	0.029	15,517	0.018	73,000	0.020
	Sangha	89	0.021	12,468	0.014	57,559	0.016
	Likouala	150	0.036	31,682	0.036	154,104	0.042
	Subtotal	1,885	0.455	337,480	0.381	1,411,939	0.382
Total		4,143	1.000	885,145	1.000	3,697,493	1.000

*Source*: General population and housing census, Congo, 2007.

### Sample size

The size N of the households in the sample was calculated using the following formula: N = e. [Z^2^*p(1 - p) / d^2^], with Z = 1.96, P the expected proportion set at 50%, the value of the 5% standard error and e = 2 corresponding to coefficient used to take into account the sampling effect. Using this formula, a sample of 384 households was obtained. Considering a minimum of 2 individuals to be tested per household, it was expected that blood would be collected from at least 768 individuals in 384 households. At the national level, taking into account the 12 departments of Congo and the availability of screening tests, we set the samples at 4,608 households and 9,216 persons for screening.

### Selection of study units

Three-stage stratified sampling was used. At the 1st level, the statistical unit was the CZ defined during the mapping work of the GPHC 2007. The sampling of CZ was done in each department. In total, 116 CZ were selected by simple random sampling method [27] with the Alea function with Excel and distributed by department regarding the weight of the initial number of CZ in each department.

In the 2nd stage, the expected households were selected and distributed by department and by CZ. The sample of households per CZ was built up step by step until the set size was reached. At the 3rd level, in each household, the survey was proposed to all persons present at the time of the visit.

Were included all individuals regardless of the age years identified for recruitment into the investigation, irrespective of known acute or prior COVID-19 infection or had vaccinated against COVID-19 who resided in households under study and who are able to give consent, or in the case of minor children, whose legal guardian is able to give consent for them. For children, the respondent to the questionnaire was the head of the household (or his/her representative). When the set number of subjects was reached, the interviewers stopped the work in the respective CZ. Refusal to give informed consent and absence from the household at the time of the visit were the criteria for non-inclusion. A total of 10,137 people were contacted in face-to-face. Of these, 9,094 agreed to complete the survey, for an acceptance rate of 90%. Non-respondents were immediately replaced by another member of the household concerned. If it was not possible to substitute in the surveyed household, the next household was used and so on.

### Data collection

Data were collected using a questionnaire based on Africa CDC standardized Generic protocol for a population-based, age- and gender-stratified sero-survey study for SARS-CoV-2 [28]. The survey questionnaire is added as Supplementary material. For on-site data collection, questionnaire was created on smartphone using the KoboCollect application. All data collected across the country were centralised in real time in the national survey database. The survey deployed 199 staff including 168 interviewers and 14 initially trained supervisors, 3 database managers, 2 cartographers, 3 researchers, 7 secretariat staff, and 2 principal investigators including an epidemiologist and a biologist. All staff involved in the survey were trained in infection control procedures. These procedures include good hand hygiene and the correct use of surgical masks to limit the risk of infection of the staff themselves when in in case of contact with infected persons and the risk of spread among other survey participants. Nevertheless, all investigators were previously tested negative for COVID-19 by PCR.

### Variables

The variables explored in this study included:

#### Socio-demographic characteristics

Department assessed in twelve modalities (all departments of Congo), gender (Male/female), age groups assessed in years, nationality (Congolese/ Other nationality), occupation assessed in six modalities: (health: nurse, midwife, physician, other health workers), in front of the public (shop, driver, tourism, education, bank), pupil/student, security forces (police, gendarmerie, military), other occupation and unemployed), marital status (in union/not in union), education level (not all education, primary, college/grammar school, university).

#### Medical history

Diseases assessed included: diabetes, high blood pressure, obesity, HIV infection, cancer, chronic renal failure, epilepsy, asthma, tuberculosis, sickle cell disease, and chronic lung disease. All these comorbidities were all self-reported by the participants, including HIV. Respondents were asked to answer if they were affected by any of these diseases. Each disease was assessed as Yes/No.

#### COVID-19 related characteristics

Assessed in six variables: contact with a confirmed COVID-19 case in the last month (Yes/No), had prior COVID-19 infection (Yes/No), has received at least one dose of vaccine (Yes/No), time since vaccination assessed in months (<3, 3–6, >6), type of vaccine received (Sinopharm, Sputnik light, Sputnik V, Johnson & Johnson, Pfizer, Moderna/AstraZeneca), and completed vaccinated (Yes/No). All information concerned the vaccination was obtained from individual vaccination cards.

#### Screening for anti-SARS-CoV-2 antibodies

All study subjects were tested for the detection of SARS-CoV-2 specific antibodies (IgM and IgG) using the RightSign COVID-19IgG/IgM rapid test cassette, Biotest Hangzhou Biotech, Co. Ltd lot COV21040001 [29]. The test qualitatively detects two types of anti-SARS-CoV 2 antibodies: IgG and IgM in blood sample collected from the fingertip. When the organism first comes into contact with the pathogen, IgM is produced. IgG is produced at a later stage and provides longer-term protection for the target organism. A positive test was defined in three possibilities: IgG positive: the infection is old; the subject was no longer ill; IgM positive or IgM positive + IgG positive: the infection was active. For any suspicion of active infection, the persons concerned were referred to the nearest laboratory offering PCR.

### Ethical issues

The purpose of the survey was explained to all individuals identified for recruitment into the survey. Informed consent was obtained by a trained member of the survey team for all individuals willing to participate in the survey. Informed consent was sought from a parent or legal guardian for all minor participants. Respondents were informed that participation in the survey was voluntary and that they were free to withdraw from the survey at any time without justification. Informed consent consisted of the request for permission to collect blood samples and epidemiological data for the purpose of this survey. Ethical approval was granted by the National Health Ethics Committee (Reference Number:0342/MESRS/IRSSA/CERSSA. Date: 8 July 2021).

### Statistical analysis

Data were checked by flat sorting of all variables. This allowed for the correction and cleaning of the file to obtain correct data which was then used to produce the statistical results tables. The seroprevalence was calculated as proportions. We calculated the overall seroprevalence of SARS-CoV-2 antibodies first, then the prevalence among vaccinated persons, and finally the prevalence among unvaccinated persons. The overall seroprevalence of SARS-CoV-2 antibodies was calculated as the number of positives tests over the number of persons tested. The seroprevalence of SARS-CoV-2 antibodies among vaccinated persons was calculated as the number of positives tests over the number of vaccinated persons tested. The seroprevalence of SARS-CoV-2 antibodies among unvaccinated persons was calculated as the number of positives tests over the number of unvaccinated persons tested. We also provided seroprevalence by age group and by department.

The proportions were compared using the chi-square test. Means and deviations were calculated for number of subjects per household and age. Logistic regression was used to identify factors associated with positive tests. First, a univariate analysis was conducted: in this part, all independent variables were crossed with the binary dependent variable (test result: positive or negative). The crude odds ratios were calculated as well as their 95% CIs. Next, a multivariate analysis was conducted. For this purpose, all variables with a *P*-value ≤0.20 were included in the model. The final multivariate model was obtained using a step-by-step selection procedure based on the log-likelihood ratio test to eliminate non-significant variables (*P* > 0.05) from the initial model. Only the final model is presented in the results. Statistical analyses were performed with IBM SPSS 23 software.

## Results

### Socio-demographic characteristics of respondents

Details on the distribution of the number of CZ, households, and respondents are shown in Table 2. A total of 116 CZ and 4,340 households were visited, and 9,094 persons were tested in the 12 departments of Congo. The average number of subjects per household was 4.05 ± 2.38 persons ranging from 1 to 20 persons. As shown in Table 3, 4,229 (46.5%) respondents were male and 4,865 (53.5%) were female. Mean of age was 30.18 ± 18.65 years ranging from 1 month to 101 years. Persons aged between 10 and 49 years represent 68.5% of the respondents. Regarding the level of education, 87.7% had at least primary school education and 12.3% had never attended school. Concerning professional occupation, pupils and students are the most frequent (36.5%) followed by the unemployed (31.5%). People engaged in activities that bring them into frequent contact with the public accounted for 13.6%, while health workers accounted for 2.5%. Hypertension (11.0%), diabetes (2.8%), and asthma (2.4%) were the most common medical problems declared by respondents in the study population.

**Table 2.** Proportion of counting zones, households and number of persons tested surveyed by department: Seroprevalence survey of SARS-CoV-2 antibodies in Congo, February 2022

Department	Number of counting zones selected	%	Number of Households surveys	%	Number of persons tested	%
Bouenza	11	9.5	355	8.2	770	8.5
Brazzaville	36	31.0	1,755	40.4	3,471	38.2
Cuvette	6	5.2	240	5.5	413	4.5
Cuvette Ouest	4	3.4	115	2.6	215	2.4
Kouilou	4	3.4	98	2.3	293	3.2
Lékoumou	4	3.4	78	1.8	237	2.6
Likouala	4	3.4	99	2.3	353	3.9
Niari	8	6.9	128	2.9	279	3.1
Plateaux	7	6.0	210	4.8	385	4.2
Pointe Noire	20	17.2	954	22.0	1,893	20.8
Pool	8	6.9	250	5.8	565	6.2
Sangha	4	3.4	58	1.3	220	2.4
Total	116	100.0	4,340	100.0	9,094	100.0

**Table 3.** Main socio-demographic characteristics and medical history of respondents: Seroprevalence survey of SARS-CoV-2 antibodies in Congo, February 2022

Variables	Number	Percentage (%)
*Gender*		
Male	4,229	46.5
Female	4,865	53.5
*Age groups (years)*		
<2	201	2.2
2–4	480	5.3
5–9	721	7.9
10–19	1,646	18.1
20–29	1,844	20.3
30–39	1,547	17.0
40–49	1,189	13.1
50–59	715	7.9
60–69	492	5.4
70–79	202	2.2
≥80	57	0.6
*Nationality*		
Congolese	8,778	965
Other	316	3.5
*Occupation*		
Health (nurse, midwife, physician, other health workers)	229	2.5
In front of the public (shop, driver, tourism, education, bank)	1,234	13.6
Pupil/Student	3,321	36.5
Security forces (police, gendarmerie, military)	56	0.6
Unemployed	2,867	31.5
Other occupation	1,387	15.3
*Marital status*		
In union	2,467	36.1
Not in union	4,364	63.9
*Education level*		
Not all education	1,112	12.2
Primary	2,137	23.5
College/Grammar school	4,460	49.0
University	1,385	15.2
*Medical history*		
Diabetes	194	2.8
High blood pressure	749	11.0
Obesity	66	1.0
HIV infection	8	0.1
Cancer	5	0.1
Chronic renal failure	14	0.2
Epilepsy	27	0.4
Asthma	163	2.4
Tuberculosis	27	0.4
Sickle cell disease	28	0.4
Chronic lung disease	17	0.2

### COVID-19 infection

Regarding past COVID-19 diagnosis, participants were asked if they had ever had a COV-19 infection since the beginning of the epidemic. A total of 37 out of 9,094 respondents answered yes, that is, 0.4%.

### Vaccination against COVID-19

As shown in Table 4, a total of 6,404 persons among respondents eligible for vaccination (aged over 15 years) were tested for SARS-CoV-2 antibodies. Among them, 1,919 were vaccinated against COVID-19 accounting for 30%. Concerning the time between the date of vaccination and the date of serological test, 35.2% of respondents among the vaccinated had received the vaccine 3 to 6 months ago and 27.7% more than 6 months ago. The most frequent vaccines were as follows: Johnson & Johnson (47.4%), Sinopharm (33.8%), Sputnik light (9.5%), Sputnik V (4.3%). It is worth noting that 4.0% of those who reported being vaccinated had no proof of vaccination (health card).

**Table 4.** Characteristics of respondents in relation to COVID-19: Seroprevalence survey of SARS-CoV-2 antibodies in Congo, February 2022

Variables	Number	Percentage (%)
*Contact with a confirmed COVID-19 case in the last month*		
Yes	114	1.7
No	6,717	98.3
*Prior COVID-19 infection*		
Yes	37	0.4
No	9,057	99.6
*Has received at least one dose of vaccine*		
Yes	1,919	30.0
No	4,485	70.0
Total	6,404	100.0
*Time since vaccination (months)*		
<3	247	12.9
3–6	675	35.1
>6	997	52.0
Total	1,919	100.0
*Type of vaccine received*		
Sinopharm^a^	649	33.8
Sputnik light^b^	183	9.5
Sputnik V^a^	82	4.3
Johnson & Johnson^b^	910	47.4
Pfizer^a^	16	0.8
Moderna/Astrazeneca^a^	3	0.2
Lack of proof of vaccination	76	4.0
Total	1,919	100.0
*Immunization status*		
Fully vaccinated	1,718	89.5
Partially vaccinated	201	10.5
Total	1,919	100.0

a two doses recommended.

b single dose recommended.

### Overall seroprevalence of anti-SARS-CoV-2 antibodies

Table 4 shows results of serological rapid tests. Among 9,094 persons tested, 4,383 tests were positive, giving an overall prevalence of 48.2% [47.2%–49.2%].

### Seroprevalence of anti-SARS-CoV-2 antibodies by vaccination status

As shown in Table 4, 6,404 persons tested were eligible for vaccination (i.e., 1,919 vaccinated and 4,485 unvaccinated) according to national guidelines, representing 81% of study respondents. Among 1,919 vaccinated persons tested, 1,366 tests were positive for a prevalence of 71.2% [69.1%–73.2%]. Among 4,485 unvaccinated persons, 1,884 tests were positive for a prevalence of 42.0% [40.9%–43.2%]. The difference between the proportion of positive tests in vaccinated and unvaccinated subjects is statistically significant (*P* < 0.001). The seroprevalence among children under age 16 (not eligible for vaccination at the time of the study) was 37.1% (Table 5).

**Table 5.** Seroprevalence of SARS-CoV-2 antibodies in Congo by immunization status, February 2022

Categories	Number of persons tested	Positive tests	Seroprevalence (%)	95% CI
All persons	9,094	4,383	48.2	47.2–49.2
Vaccinated persons	1,919	1,366	71.2	69.1–73.2
Unvaccinated persons eligible for vaccination	4,485	2,018	45.0	43.5–46.5
Unvaccinated persons not eligible for vaccination (children under 16 years)	2,690	999	37.1	35.5–39.0

### Seroprevalence of anti-SARS-CoV-2 antibodies by age

Table 6 shows the distribution of people with anti-SARS-CoV-2 antibodies in Congo as of February 2022, by age group. Among those tested, test results were most frequently positive in people aged 20–29 years (50.8% [48.5%–53.0%].), 30–39 years (50.0% [47.5%–52.5%]), 40–49 years (54.9% [51.1%–57.1%].), 50–59 years (57.6% [54.0%–61.2%].), 60–69 years (60.0% [55.6%–64.2%].), 70–79 (55.9% [48.8%–62.9%]).

**Table 6.** Seroprevalence of anti-SARS-CoV-2 antibodies in Congo by age, February 2022

Age (years)	Number of persons tested	Positive tests	Seroprevalence (%)	95% CI
<2	201	66	32.8	26.4–39.8
2–4	480	141	29.4	55.5–33.6
5–9	721	226	31.3	28.1–34.8
10–19	1,646	744	45.2	42.8–47.6
20–29	1,844	936	50.8	48.5–53.0
30–39	1,547	774	50.0	47.5–52.5
40–49	1,189	653	54.9	52.1–57.7
50–59	715	412	57.6	54.0–61.2
60–69	492	295	60.0	55.6–64.2
70–79	202	113	55.9	48.8–62.9
≥80	57	23	40.4	27.6–54.2

### Seroprevalence of anti-SARS-CoV-2 antibodies, by department

**Figure 1.**
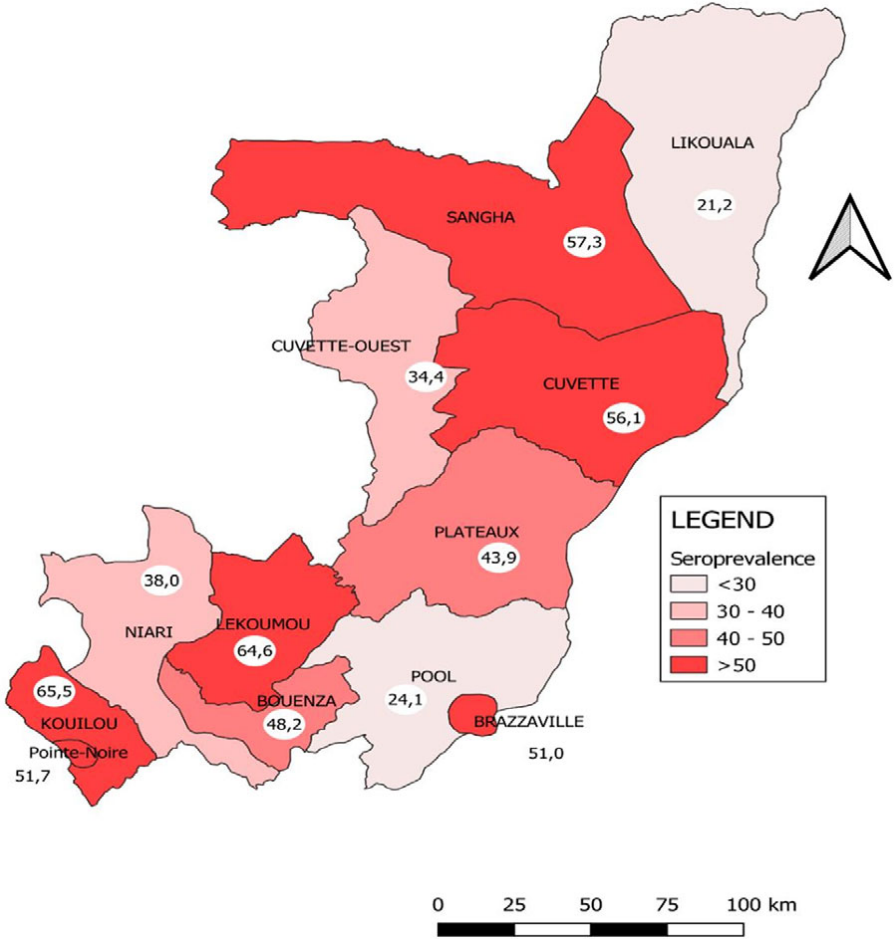
Distribution of SARS-CoV-2 antibodies seroprevalence by department in Congo, February 2022.

Figure 1 illustrates the distribution of the frequency of anti-SARS-CoV-2 antibodies in the twelve administrative departments of the Republic of Congo in February 2022. Two departments have reached herd immunity (seroprevalence of SARS-CoV-2 antibodies greater than or equal to 60%): Kouilou (65.5% [59.8–71.0]) and Lékoumou (64.6% [58.1–70.6]). Departments of Sangha (57.3% [50.5–63.9]), Cuvette (56.2% [51.4–60.9]), Pointe Noire (51.7% [49.5–54.0]), and Brazzaville (51.0% [49.3–52.7]) approach herd immunity. Six departments have low prevalence rates, that is, Plateaux (43.9% [39.0–48.9] Niari (38.0% [32.3–44.0]), Cuvette Ouest (34.4% [28.1–41.2]), Pool (24.1% [20.7–27.8]), and Likouala (21.2% [17.3–25.8]).

### Factors associated with SARS-CoV-2 antibody positivity

In univariate analysis, as shown in Table 7, the variables significantly associated with a positive serological test were as follows: having been vaccinated, respondent’s age, occupation, marital status, level of education, contact with COVID-19 patients, alcohol consumption, presence of comorbidities, and history of travel in the last month prior to serologic test. In multivariate analysis, being vaccinated (OR: [95% CI]): (3.01 [2.66–3.41]), having been in contact with a COVID-19 patient: (2.14 [1.35–3.38]), being over 50 years of age: (1.36 [1.19–1.55]), living in a union: (1.13 [1.01–1.26]) having secondary education: (1.47 [1.18–1.84]), having tertiary education: (1.48 [1.15–1.90]) were factors independently associated with a higher risk of having anti-sars-CoV-2 antibodies. Conversely, having an occupation with public exposure (OR [95% CI] =0.67 [0.49–0.93]) was significantly associated with a lower risk of having anti-SARS-CoV-2 compared to health workers.

**Table 7.** Factors associated with severe acute respiratory syndrome coronavirus-2 (SARS-CoV-2) antibody positivity in Congo: Seroprevalence survey of SARS-CoV 2 antibodies in Congo, February 2022

Variables	Positive tests	Negative tests	OR _crude_ [IC95%]	*P*-value	OR _adjusted_ [CI95%]	*P*-value
Has received at least one dose of vaccine						
Yes	1,366 (71.2)	553 (28.8)	3.40 [3.05–3.80]	0.000	3.01 [2.66–3.41]	0.000
No	3,017 (42.0)	4,158 (58.0)	1			
Gender						
Male	2,004 (47.1)	2,225 (52.6)	0.94 [0.87–1.02]	0,150	0.76 [0.69–0.85]	0.000
Female	2,379 (48.9)	2,486 (51.1)				
Age groups (years)				0.000		
<18	999 (37.1)	1,691 (62.9)	0.56 [0.51–0.61]	0.000	0.95 [0.76–1.19]	0.686
18–49	2,541 (51.5)	2,397 (4.5)	1			
≥50	843 (57.5)	623 (42.5)	1.28 [1.35–1.44]	0.000	1.36 [1.19–1.55]	0.000
Professional occupation						
Health (nurse, midwife, physician, other health workers)	164 (71.6)	65 (28.4)	1			
In front of the public (shop, driver, tourism, education, bank)	644 (52.2)	590 (47.8)	0.43 [0.32–0.59]	0.000	0.67 [0.49–0.93]	0.017
Pupil/student	1,535 (46.2)	1,786 (53.8)	0.34 [0.25–0.46]	0.000	1.01 [0.73–1.40]	0.95
Security forces (police, gendarmerie, military)	38 (67.9)	18 (32.1)	0.84 [0.45–1.57]	0.579	0.76 [0.40–1.46]	0.417
Unemployed	1,292 (45.1)	1,575 (54.9)	0.32 [0.24–0.44]	0.000	0.77 [0.56–1.06]	0.114
Other occupation	710 (51.2)	677 (48.8)	0.42 [0.31–0.56]	0.000	0.76 [0.55–1.06]	0.102
Marital status						
Yes	1,362 (55.2)	1,105 (44.8)	1.20 [1.08–1.32]	0.000	1.13 [1.01–1.26]	**0.03**
No	2,214 (50.7)	2,150 (49.3)				
Education level						
Not all education	386 (34.7)	726 (65.3)	1			
Primary	887 (41.5)	1,250 (58.5)	1.33 [1.15–1.55]	0.000	1.24 [0.97–1.59]	0.087
College/grammar school	2,294 (51.4)	2,166 (48.6)	1.99 [1.74–2.28]	0.000	1.47 [1.18–1.84]	0.001
University	816 (58.9)	569 (41.1)	2.70 [2.29–3.18]	0.000	1.48 [1.15–1.90]	0.002
Contact with a confirmed COVID-19 case in the last month						
Yes	88 (77.2)	26 (22.8)	3.13 [2.02–4.87]	0.000	2.14 [1.35–3.38]	0.001
No	3,488 (51.9)	3,229 (48.1)	1			
Alcohol consumption						
Yes	1,771 (55.2)	1,440 (44.8)	1.34 [1.12–1.36]	0.000	1.19 [1.07–1.32]	0.001
No	1,805 (49.9)	1,815 (50.1)	1			

## Discussion

In this study, we assessed the level of circulation of the COVID-19 virus in Congo two years after the detection of the first case, by detecting specific anti-sars-cov-2 antibodies in a national representative sample. This is the first study addressing the prevalence of SARS-COV-2 in Congo. Results show that a large proportion of the population has already been in contact with the Covid-19 virus, in all age groups and departments of the country. Some departments have reached the expected level of herd immunity corresponding to an antibody prevalence of 60%.

One of the main findings of this study is the high level of SARS-CoV2 antibodies in both vaccinated and unvaccinated subjects. With an overall prevalence of 48%, based on a population of 5,781,511, we estimate that 2,786,688 persons had antibodies against SARS-CoV-2 in February 2022 (Table 7). When restricted analysis to unvaccinated persons, the prevalence is 42%, corresponding to 1,164,803 persons who had antibodies against COVID-19 based on a population of 2,468,907 unvaccinated people. Our estimations are well above the cumulative incidence of 24,690 confirmed cases reported by the Government as of 18 July 2022, corroborating the findings of the meta-analysis study conducted by Lewis et al. which reported a high prevalence of SARS-CoV-2 in Africa between 2020 and 2021 and large under-ascertainment of infection based on confirmed case-based data [30]. These results are very interesting, as they support that the COVID-19 virus has circulated strongly in the country without any pressure on the health services. Three possible explanations can support the paradox between the official cases and the results of our study. The first explanation is the low capacity for mass screening, and only 441,380 were tested in the whole country. The second explanation is the frequent use of traditional therapists by the population [31, 32]. It is known that the Ministry of Health does not take into account the active files of these practitioners in official statistics. Finally, the third explanation is the fact that the majority of people who contract the virus are often asymptomatic and therefore remain outside the national data collection system [33]. This statement on the paradox was reported in previous population-based studies conducted on non-representative samples in Congo [12, 14]. Regardless of the vaccination status of those screened, our prevalence results corroborate those of serological surveys with representative samples conducted in other African countries [7, 34, 35]. However, national prevalence differs from country to country [30], due to several parameters such as the number of outbreaks to which populations have been exposed, the number of people already vaccinated, and the kinetic variation of antibodies over time. The high prevalence observed in Congo in February 2022 can be explained by the fact that the country has experienced four outbreaks since the official notification of the first case of COVID-19 on 14 March 2020 [36]. Our results show that epidemiological surveillance systems based on PCR tests, in a context of limited resources, do not allow us to know the real situation of the COVID-19 epidemic. Indeed, the majority of tests only concern travellers and contacts of people infected with Covid-19. In this context, in order to strengthen PCR screening, it is important to extend the collection of samples to people with symptoms of the disease in health facilities and the offices of traditional and religious practitioners.

In Congo, two departments out of twelve are epicentres of COVID-19, namely Brazzaville and Pointe-Noire. These two departments account for more than 80% of officially reported cases in the country. In these two departments, the proportions of people who had antibodies exceeded 50%, since the majority of people vaccinated are located there. It can be observed that the proportions obtained in the other departments are due to natural immunity, as few people have been vaccinated in these departments. In the departments with low prevalence and a low proportion of people vaccinated, such as Pool, Likouala, and Cuvette Ouest, it is clear that the COVID-19 virus has circulated very little. These are departments that are either far from urban centres or difficult to access. Consequently, these departments with low prevalence of SARS-COV-2 antibodies are not immune to outbreaks.

According to the WHO [37], In children and adolescents, SARS-CoV-2 infection generally results in milder disease, which may result in less testing and therefore under-detection of SARS-CoV-2 infection in children and adolescents. In Congo, official estimations show 1,558 registered COVID-19 cases among people under 20 years of age since the start of the pandemic. With an antibody prevalence of 42.1%, we estimate that 973,606 persons of age less than 16 years have already been infected with SARS-CoV-2 based on a population of 2,312,604. Similar prevalence trends have been observed in other African countries [7].

As expected, vaccination is a factor associated with the presence of SARS-CoV-2 antibodies. Indeed, vaccinated individuals were more likely to have antibodies than unvaccinated individuals, as reported [7]. However, it is impossible in this study to say whether the presence of antibodies precedes or follows the vaccination. Indeed, the cross-sectional design of our study does not allow this. Nevertheless, our results support a beneficial effect of vaccination in the production of antibodies [38, 39]. However, we do not know if these antibodies have a beneficial effect in terms of protection against infection and against severe disease because the tests used in this study are not appropriate to give this precision. Nonetheless, the limited number of hospitalizations for COVID-19 observed in the country may be related to the fact that those infected had neutralizing antibodies against SARS-COV-2.

Unexpectedly, our results show that the seroprevalence among children was lower than among adults. This can be explained by the fact that in Congo, children are not targeted by COVID-19 vaccination program. Our findings show that seroprevalence is very high among vaccinated people in comparison with no vaccinated people. In addition, the schools, places of mixing, and possible source of contaminations of the children were closed during the periods of confinement, at the time of the epidemic waves.

Our results also show that the type of occupation is a factor associated with the risk of contracting the covid-19 virus. Indeed, ‘public exposure’ occupations were significantly associated with a lower risk of having anti-SARS-CoV-2 compared to health workers. In this study, ‘public exposure’ refers to group of occupations that are frequently exposed to people. The following occupations were included in this group: shop, driver, tourism, education, and bank. We know that health workers are more likely to encounter covid-19 patients in comparison with the first group. This is a possible reason for this difference in risk of having antibodies between the two groups. People who work mainly in closed environments are more exposed to the risk of contracting the virus than those who work in more open environments. In this sense, our results support preventive measures based on not requiring the wearing of protective masks in open environments.

In this study, a probable bias of classification linked to the use of serologic tests must be taken into account when considering the prevalence obtained. Indeed, the serological test used in this study is neither 100% sensitive nor specific [40]. It would have been interesting to confirm the results of positive tests by [41, 42] PCR in order to identify the true-positives among persons suspected of having an active infection (IgM positive or IgM positive + IgG positive).

In conclusion, our study shows that government statistics underestimate the magnitude of COVID-19 in the Congo. We estimate that in February 2022 48% persons had antibodies against the COVID-19 virus, 45% unvaccinated persons, and 37.1% persons not eligible for vaccination (children and adolescents under 16 years of age) have already experienced infection by the SARS-CoV-2. Vaccination is playing an important role in antibody production and its intensification in low prevalence departments will reduce the risk of new outbreaks.

## Supporting information

Ndziessi et al. supplementary material Ndziessi et al. supplementary material

## Data Availability

Readers can contact the authors if they want access to data.

## Supplementary material

For supplementary material accompanying this paper visit http://doi.org/10.1017/S0950268823001425. click here to view supplementary material
