# Supplementary material for: Seroprevalence of SARS-CoV-2 antibodies in Republic of Congo, February 2022
Source: Epidemiol Infect. 2023 Sep 11;151:e162. doi: 10.1017/S0950268823001425 (PMC10600732; doi:10.1017/S0950268823001425)
Supplement: Ndziessi et al. supplementary material [file S0950268823001425sup001.docx]

**^Appendix 1. SURVEY QUESTIONNAIRE^**

| **SEROEPIDEMIOLOGIC SURVEY STRATIFIED BY AGE GROUP ON INFECTION**  **WITH CORONAVIRUS 2019 (COVID-19) IN CONGO** |
| --- |

**^QUESTIONNAIRE^**

**Date of data collection**: I____I____I/ I____I____I/ I____I____I____I____I

**Identification Number:**

Identifier (first 3 letters Last name then first 3 letters First name, Gender (M/F), Year of birth):

I____I____I____I____I____I____I____I____I____I____I____I

**Sample ID no:** ……………………………………………………………………………………………………

**Department:** …………………………………………………………………………………………………….

ZD: ……………………………………………………………………….

Urban/Rural: □ Urban □ Rural

1. **SOCIO-DEMOGRAPHIC CHARACTERISTICS**

**Q101**. Age (years): I___I___I

**Q102**. Gender: ❑ Male1 ❑ Female2

**Q103**. Nationality: ❑ Congolese1 ❑ Other2 (Specify): …………………………………………………….

**Q104**. Occupation:

□ Health (nurse, doctor, hospital staff)

□ Facing the public (store, driver, tourism, education, bank)

□ Student (elementary or secondary)

□ Unemployed

□ Other (specify)

**Q105.** Marital status: ❑ Single1 ❑ Married2 ❑ Divorced3 ❑ Widowed4

**Q106.** Education level: ❑ No schooling0 ❑ Primary1 ❑ Secondary2 ❑ Higher3

1. **EPIDEMIOLOGICAL LINK**

**Q201**. Notion of travel abroad in the last 03 months: ❑ Yes1 ❑ No0

**Q102**.1. If yes, date of return to Congo: I____I____I/ I____I____I/ I____I____I____I____I

**Q202**. Notion of contact with a confirmed covid-19 positive person: ❑ Yes1 ❑ No0

**Q202**.1. If Yes, relationship to this person: …………………………………………………………….

**Q203**. How many people do you live with in your household? I____I____I

**Q204**. How many people are you sharing the room with? I____I____I

**3. MEDICAL HISTORY AND ADDICTIVE BEHAVIORS**

**Q401**. **Medical History**

❑ Diabetes ❑ High blood pressure ❑ Obesity ❑ HIV infection ❑ Cancer ❑ Chronic renal failure ❑ Epilepsy ❑ Stroke ❑ Asthma ❑ Tuberculosis ❑ Sickle cell disease ❑ Chronic lung disease

❑ coronary heart disease, heart failure, heart attack or other serious heart disease.

❑ Other (specify): ………………………………………

**Q402.** **Addictive behaviors**

**Q402**.1. Alcohol (AUDIT-C score)

**Q402**.1.1. How often do you drink alcohol?

❑ Never0 ❑ Once a month at least1 ❑ 2 to 4 times a month2 ❑ 2 to 3 times a week3

❑ At least 4 times a week4

**Q402**.1.2. How many drinks containing alcohol do you have on a typical drinking day?

❑ <30 ❑ 3 or 41 ❑ 5 or 62 ❑ 7 to 93 ❑ 10 or more4 ❑ Not applicable5

**Q402**.1.3. How often do you have six or more drinks on a particular occasion?

❑ Never0 ❑ Less than once a month1 ❑ Once a month2 ❑ Once a week3

❑ Every day or almost4 ❑ Not applicable5

**Q402**.2. Tobacco

**Q402**.2.1. Do you currently smoke tobacco?

❑ Every day1 ❑ Less than once a day2

❑ Not at all (no)3 (if not at all or no answer go to question Q402.2.3)

❑ No response99

**Q402**.2.2. Since when do you smoke every day

❑ Age of beginning of tobacco (years): I___I___I Or Number of years since the beginning I___I___I

**4. SYMPTOMS**

**Q501.** Have you ever tested positive for COVID-19: □ Yes □ No

**a**. If No, go to question 1 about vaccination

**b**. If yes, by which test? □ PCR □ rapid antigen test □ rapid serological test □ Don't know

**C.** If yes, what is the date of diagnosis: day………../month…………/year 202….

**d**. If yes, have you had any of the following symptoms in association with your diagnosis of COVID-19?

Fever ≥38°C: □ Yes □ No □ Unknown

Chills: □ Yes □ No □ Don't know

Fatigue: □ Yes □ No □ Don't know

Muscle pain (myalgia): □ Yes □ No □ Unknown

Sore throat □ Yes □ No □ Don't know

Cough: □ Yes □ No □ Don't know

Runny nose: □ Yes □ No □ Unknown

Shortness of breath: □ Yes □ No □ Don't know

Wheezing: □ Yes □ No □ Don't recall

Chest pain: □ Yes □ No □ Unknown

Headache: □ Yes □ No □ Don't know

Loss of sense of smell: □ Yes □ No □ Unknown

Loss of taste: □ Yes □ No □ Don't know

Diarrhea: □ Yes □ No □ Don't know

Other symptoms:

e. Date of onset of symptoms: day………../month…………/year 202….

**5. IMMUNIZATION**

1. Have you been vaccinated against COVID-19? □ Yes □ No

2. If yes, what vaccine did you receive? ___________________________________

3. What is the date of vaccination?

4. How many doses did you receive?

**THANK YOU**
